# Challenges and Opportunities: Studying CKDu in the United States

**DOI:** 10.34067/KID.0000000000000408

**Published:** 2024-03-06

**Authors:** Sophie E. Claudel, Maya Chan, Madeleine K. Scammell, Sushrut S. Waikar

**Affiliations:** 1 Department of Medicine, Boston Medical Center, Boston University Chobanian & Avedisian School of Medicine, Boston, Massachusetts; 2 Boston University Chobanian & Avedisian School of Medicine, Boston, Massachusetts; 3 Department of Environmental Health, Boston University School of Public Health, Boston, Massachusetts; 4 Section of Nephrology, Department of Medicine, Boston Medical Center, Boston University Chobanian & Avedisian School of Medicine, Boston, Massachusetts

**Keywords:** CKD, clinical epidemiology, minority health and disparities, social determinants of health, USRDS (United States Renal Data System)

CKD of unknown etiology (CKDu) predominantly affects young adults in rural Central America and southern Asia with devastating effects on individuals and communities. Unlike most CKD in the United States, CKDu is not caused by or preceded by traditional risk factors, including hypertension or diabetes. Diagnosis often occurs at late stages, and treatment is difficult or impossible to access in affected communities. Most investigations of CKDu focus on individuals residing in endemic communities; in contrast, little research explores the occurrence of CKDu in other countries. There are several possible reasons why CKDu may exist in the United States. First, there may be common environmental exposures leading to CKDu in the United States and other endemic regions. Second, CKDu may arise from distinct exposures that nevertheless have similar downstream effects on kidney function. Finally, given immigration patterns in the Americas, individuals with CKDu may migrate to the United States.

## Efforts to Characterize Kidney Function among Latinx Populations in the United States as a Surrogate for CKDu

Given the correlation between CKDu and agricultural work in endemic communities, research in the United States has focused on kidney function in Latinx farmworkers. One hypothesis is that occupational exposure to extreme heat stress leads to recurrent AKI, resulting in CKDu. A study of predominantly Latinx agricultural workers in Florida found that heat index was significantly associated with odds of AKI after multivariable adjustment for individual characteristics.^1^ Similarly, among a small cohort of Latinx agricultural workers in California, 11.8% developed post-shift AKI.^2^ Risk of AKI was higher among workers paid by the piece, a payment system that may incentivize greater intensity work with fewer breaks, which is common in endemic areas of Central America.^2^ Neither of these studies has been extended to study CKDu specifically, which would require longitudinal follow-up and further clinical investigation.

Another approach to characterizing CKD in the United States has been to study incident ESKD in relation to potentially shared environmental exposures with CKDu endemic regions. A California-based study found that geographical regions with an over-representation of ESKD had a higher proportion of unexplained cause of ESKD compared with other areas of the state; additionally, these regions had higher groundwater nitrate levels.^3^ In this study, unexplained cause included ESKD attributed to hypertension, interstitial nephritis, and unknown cause, on the basis of data from the United States Renal Data System (USRDS) between 2015 and 2017.^3^ There are several potential sources of bias in this approach. It assumes that non-diabetic and non-glomerular disease are consistent with a diagnosis of CKDu; however, substantial misclassification exists when using administrative data for ascertaining the presumed cause of ESKD.^4^ In addition, patients are typically not recorded in USRDS unless they receive outpatient dialysis for at least 45 days, which excludes patients who return to their country of origin after diagnosis. Finally, the analyses could not adjust for confounding related to alternative exposures or individual medical comorbidities because of the limitations of USRDS.

## The Challenges of Identifying CKDu among Undocumented Immigrants in the United States

CKDu may be more prevalent and identifiable among patients receiving emergency-only dialysis than in USRDS. Undocumented immigrants in the United States cannot receive routine outpatient dialysis care in most US states and rely on emergency dialysis; these patients are not reported to USRDS.^5^ An abstract from the 2023 World Congress of Nephrology highlighted that patients receiving recurrent emergency dialysis in Texas were younger and more likely to have an unknown cause of ESKD as compared with average individuals in USRDS.^6^ The authors estimate that between 1% and 17% of the patients identified may have CKDu on the basis of absent traditional CKD risk factors. However, the analysis is limited by lack of race/ethnicity, detailed clinical phenotype, or occupational data.^6^

**Figure 1:**
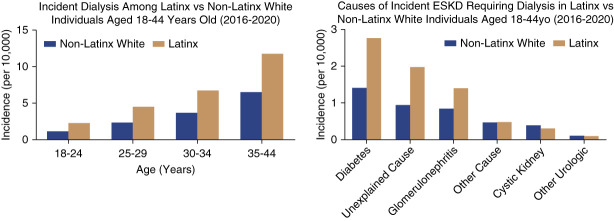
**Dialysis initiation by ethnicity, age, and cause of ESKD in the United States.** Data from the USRDS between 2016 and 2020 showing (A) higher incidence of dialysis in Latinx adults aged 18–44 years and (B) greater proportion of adults with unexplained ESKD are Latinx compared with non-Latinx White. USRDS, United States Renal Data System.

We reviewed USRDS data on dialysis initiation by attributed cause and demographic characteristics. Between 2016 and 2020, Latinx individuals (listed as “Hispanic” in USRDS) initiated dialysis approximately 6.7 years earlier than non-Latinx White adults and 1.1 years earlier than non-Latinx Black adults. The incidence of dialysis initiation among those aged 18–44 years was 1.9 times higher among Latinx than non-Latinx White adults (Figure 1A). Using a definition of unexplained ESKD, including hypertension-attributed and unknown causes,^3^ proportionally more adults with unexplained ESKD were Latinx than non-Latinx White (Figure 1B). These analyses likely underestimate the true burden of unexplained ESKD given the exclusion of adults receiving emergency-only dialysis, which remained the sole source of dialysis care for undocumented immigrants in 30 states by 2019. They are additionally prone to the same biases as others relying on USRDS to examine potential CKDu.

## Community Perceptions of CKDu in the United States

Kidney disease awareness is high in CKDu-endemic regions. However, it is unknown how individuals from affected communities view their risk upon immigration to the United States. They may continue to be at risk of the disease through underlying genetic predisposition, preimmigration environmental or occupational exposure, or ongoing, postimmigration exposure to agrochemicals, heat stress, allostatic load, or environmental pollutants (*e.g*., microplastics, heavy metals, per- and polyfluoroalkyl substances). Three quarters of farmworkers in the United States are Latinx immigrants.^7^ Latinx children as young as 10 years are known to work on farms in North Carolina, where they are at increased risk of heat-related illness.^8^ Ethnographic work profiling Latinx farmworkers in California suggests a similar phenotype to CKDu and rising awareness of the disease.^9^ Intense pressure from field supervisors and the competitive environment of day labor contribute to conditions that may increase risk of AKI.^9^ Clearly not all Latinx immigrants are employed in agricultural occupations, and CKDu is also observed in non-agricultural workers in Central America. Further work is urgently needed to understand both the perceived and measurable risk in first-generation immigrants and their families.

## Opportunities for Future Research

One of the challenges of studying CKDu in the United States is the definition of CKDu itself, an ill-defined collection of exclusion criteria that says more about what we know the disease is not than what it is. Patients with CKDu may have their first contact with medical care late in the disease course after they have developed comorbidities and when biopsy is less likely to be performed. Thus, a patient with CKDu could inadvertently be labeled with hypertensive nephropathy or even diabetic kidney disease when the kidney pathology occurred years prior. Establishing a set of diagnostic criteria that could be applied to existing epidemiologic cohorts and administrative data would be a useful step forward but may not be sufficient. Even in states with provisions granting health insurance to undocumented immigrants, patient reluctance to seek medical care because of job insecurity or the broader sociopolitical environment may exacerbate under-recognition of CKDu.^9^

National datasets yield limited opportunity to determine the extent of CKDu in the United States. The National Health and Nutrition Examination Survey affords a nationally representative population of Latinx adults, but because of the cross-sectional design, it is not possible to determine whether diagnosis of comorbidities preceded a decline in eGFR. Moreover, identifying CKD in the National Health and Nutrition Examination Survey relies on eGFR alone, which is not specific or sensitive for CKDu. Excluding individuals with traditional comorbidities introduces assumptions regarding the temporal relationship of these conditions but offers a preliminary view of possible CKDu at the population level.

There are several approaches that could expand our understanding of CKDu in the United States, including developing longitudinal cohorts of individuals hypothesized to be at risk of CKDu. Cohorts could be developed on the basis of occupational, environmental, or geographic risk (*e.g*., agricultural workers in high-heat areas) or by exploring disease prevalence in community-based health clinics that serve predominantly Latinx populations. Explorations of CKDu in the United States would benefit from deep phenotyping approaches, including incorporation of multiomics platforms, kidney biopsy, and environmental monitoring that are increasingly being used to study the disease in endemic regions. Community-based studies would also allow for complementary qualitative research on patient and provider perspectives of CKDu. While the existing literature assessing CKDu in the United States focuses on the Latinx community, CKDu is also present in communities across India, Sri Lanka, and potentially Nepal. Future work should remain vigilant to not overlook these populations. Unfortunately, the epidemiologic study of Asian adults in the United States is hampered by a distinct lack of ancestry reporting.

In conclusion, whether CKDu is present in the United States because of shared environmental exposures with traditionally endemic regions or because of global migration of susceptible individuals, the disease is understudied and likely under-recognized in the United States. The nephrology and public health workforces must use anticolonial research strategies that allow the voices of these vulnerable individuals to be heard and for the benefits of the findings to be felt by those most in need.^10^ Such strategies include incorporation of community-based participatory research methods and developing collaborations with in-country investigators from endemic regions to understand global patterns of disease. The devastation of CKDu may extend beyond borders because of immigration patterns, global warming, and largely unexplored environmental nephrotoxins. Methodologically sophisticated research and activism will be required to alleviate the effect of CKDu on global health.
